# Targeting the Innate Immune Kinase IRAK1 in Radioresistant Cancer: Double-Edged Sword or One-Two Punch?

**DOI:** 10.3389/fonc.2019.01174

**Published:** 2019-11-13

**Authors:** Peter H. Liu, Samuel Sidi

**Affiliations:** ^1^Division of Hematology and Medical Oncology, Icahn School of Medicine at Mount Sinai, Tisch Cancer Institute, New York, NY, United States; ^2^Department of Cell, Developmental and Regenerative Biology, The Graduate School of Biomedical Sciences, Icahn School of Medicine at Mount Sinai, New York, NY, United States; ^3^Department of Oncological Sciences, Icahn School of Medicine at Mount Sinai, New York, NY, United States

**Keywords:** IRAK1, irak4, radiation therapy, immunotherapy, radiosensitiser

## Abstract

Antitumor immunity has emerged as a favorable byproduct of radiation therapy (RT), whereby tumor-associated antigens released from irradiated cells unleash innate and adaptive attacks on tumors located both within and outside the radiation field. RT-induced immune responses further provide actionable targets for overcoming tumor resistance to RT (R-RT); immunotherapy (IT) with checkpoint inhibitors or Toll-like receptor (TLR) agonists can markedly improve, if not synergize with, RT in preclinical models, and several of these drugs are currently investigated as radiosensitizers in patients. In an unbiased chemical-genetic screen in a zebrafish model of tumor R-RT, we unexpectedly found that Interleukin 1 Receptor-Associated Kinase 1 (IRAK1), a core effector of TLR-mediated innate immunity, also functions in live fish and human cancer models to counter RT-induced cell death mediated by the PIDDosome complex (PIDD-RAIDD-caspase-2). IRAK1 acting both as a driver of intrinsic tumor R-RT and as an effector of RT-induced antitumor immunity would, at first glance, pose obvious therapeutic conundrums. IRAK1 inhibitors would be expected to sensitize the irradiated tumor to RT but simultaneously thwart RT-induced antitumor immunity as initiated by stromal dendritic cells. Conversely, TLR agonist-based immunotherapy would be expected to intensify RT-induced antitumor immunity but at the expense of fueling IRAK1-mediated cell survival in the irradiated tumor. We discuss how IRAK1's differential reliance on catalytic activity in the radiation vs. TLR responses might help overcome these hurdles, as well as the crucial importance of developing IRAK1 inhibitors that lack activity against IRAK4, the kinase activity of which is essential for IRAK1 activation in both pathways.

## IRAK1: a Core Effector in IL-1R/TLR Innate Immune Signaling

IRAK1 is a conserved death domain (DD)-containing protein kinase whose *Drosophila* homolog, pelle, transduces dorso-ventral patterning and microbial cues recognized by the transmembrane receptor, Toll (1–6). The discovery of a Toll-like receptor (TLR) family of proteins in humans (3), composed of 10 TLRs, was soon followed by the finding that, as in flies, TLRs are responsible for the innate response to microbial infection through binding to *p*athogen- and *d*amage-associated molecular patterns (PAMPs and DAMPs) and viral/bacterial nucleic acids in the intracellular space (endosomal TLRs). These discoveries were awarded the 2011 Nobel Prize in Physiology or Medicine (3).

**Figure 1:**
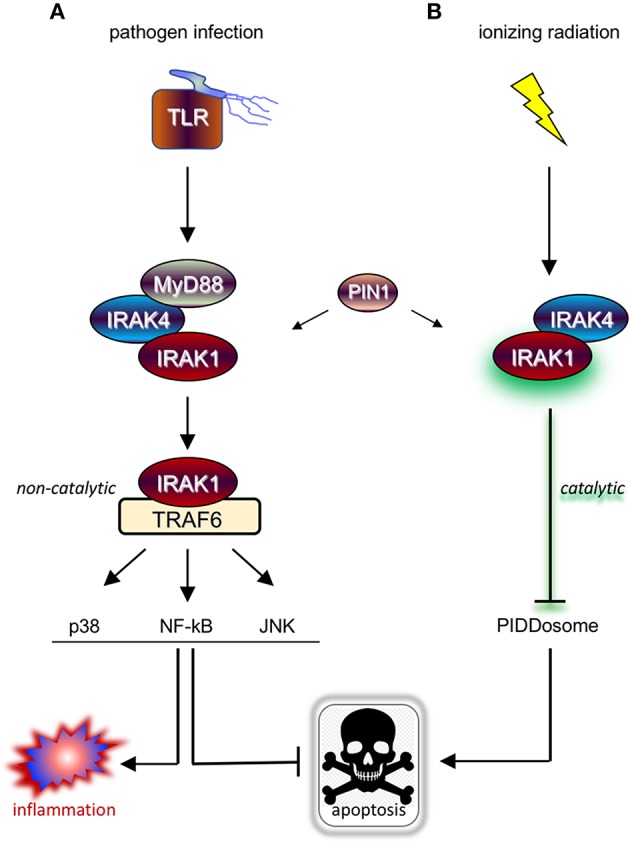
IRAK1 kinase drives distinct prosurvival responses to microbial infection and ionizing radiation. **(A)** Diagram of the TLR signaling cascade which stimulates immune cell survival and inflammation in response to pathogen sensing. Ligated TLRs recruit MyD88 to trigger Myddosome (MyD88-IRAK4-IRAK1) formation, resulting in the activation of IRAK1 and release of the kinase from the complex. In turn, the activated form of IRAK1 binds TRAF6 to enable TRAF6-mediated activation of multiple pathways involved in anti-apoptotic and pro-inflammatory signaling. **(B)** Diagram of the newly identified IRAK1 signaling pathway triggered by IR, which involves IRAK4 but not MyD88 and antagonizes apoptosis through a different route involving inhibition of PIDDosome formation. Note that while IRAK1 catalytic activity is required in the radiation response (as symbolized by a green glare), it is dispensable for microbial responses relying on TRAF6 as signaling intermediate downstream of IRAK1.

Upon ligation, TLRs and IL-1R receptor (IL-1R/TLR) signal proinflammatory and cell survival responses, the majority of which through IRAK1/4 kinases and attendant downstream signaling cascades such as NF-kB, p38/MAPK, and JNK (3, 7) (Figure 1A). IRAK1 and IRAK4 are recruited to the ligated receptor by the Toll/IL-1R homology (TIR) and DD-containing adaptor protein, Myeloid Differentiation Primary Response 88 (MyD88) (8). MyD88 engages in homotypic TIR:TIR and DD:DD interactions with IL-1R/TLR and IRAK1/4, respectively, mobilizing the kinases to the receptor and resulting in the formation of the “MyDDosome” (9) complex (MyD88-IRAK4-IRAK1) (10) (Figure 1A). Only once in the MyDDosome, comprising six MyD88, four IRAK4, and four IRAK1 subunits (11), can IRAK4 dimerize. This proximity-induced dimerization of IRAK4 is the key initiating step in IRAK1 activation, with most (10, 12–16) but not all (17) models involving *trans* autophosphorylation of IRAK4 and ultimately phosphorylation of T209 on IRAK1 by fully active IRAK4. Once primed for activation by T209 phosphorylation, IRAK1 autophosphorylates on T387 in its activation loop, resulting in full activation, dissociation from the complex, and activation of downstream pathways (Figure 1A) (10, 13). IRAK1 activation also notably involves the peptidyl prolyl *cis/trans* isomerase PIN1, whose binding to IRAK1 is required for activation within, and dissociation from, the MyDDosome, and is overall essential for TLR signaling (Figure 1A) (18). Surprisingly, whether the catalytic activity of IRAK1 is required at *any* step for its function remains unclear (5, 17, 19), with genetic studies involving kinase-dead variants questioning reliance on catalytic activity (4, 6, 19–23). Consistent with this, engagement of three major signaling branches downstream of IRAK1, namely NF-κB, p38/MAPK, and JNK, relies on physical contact between activated IRAK1 and TNF receptor-associated factor 6 (TRAF6), independently of IRAK1 catalytic activity (Figure 1A) (3, 4, 21, 24). The relative importance of catalytic vs. structural functionalities of IRAK1 is an important consideration for the development of IRAK1 inhibitors for clinical use, particularly in radioresistant cancer, and will be discussed in detail in the closing sections of this review.

## IL-1R/TLR Signaling Contributes to RT-induced Antitumor Immunity and Defines a Target for RT+IT-based radiosensitization Strategies

**Figure 2:**
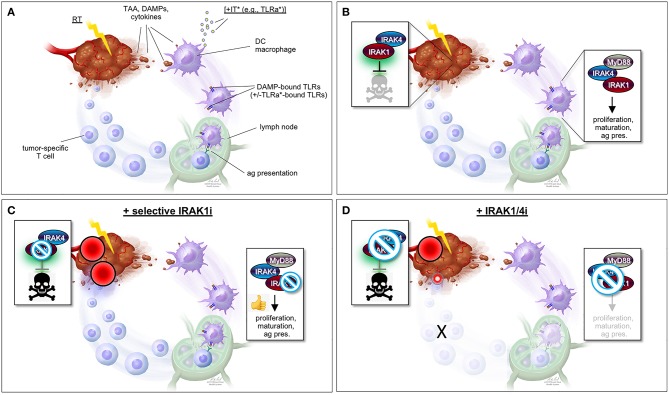
“One-two punch” vs. “double-edged sword” scenarios for tumor radiosensitization strategies exploiting IRAK1 inhibitors. **(A)** Simplified view of RT-induced antitumor immunity. DAMPs and cytokines (i.e., IL-1β) released by irradiated tumor cells are recognized by cell surface IL-1R/TLRs on surrounding stromal DCs and macrophages, stimulating their activation, maturation, and antigen presentation activity toward T-cells in lymph nodes, and ultimately unleashing tumor-specific T-cells against the irradiated tumor (as well as distant tumors not pictured here). TAA, tumor-associated antigen; DAMPs, damage-associated molecular patterns; IT, immunotherapy; TLRa, toll-like receptor agonist; DC, dendritic cell; ag pres., antigen presentation. ^*^IT (with TLRa) is optional and acts as a boost for the immune events otherwise described in the figure. **(B)** Simplified views of the IRAK1-mediated response to RT (left; tumor cell-intrinsic antiapoptotic response) and DAMP-bound TLRs (right, innate immune response). Note that while IRAK1 catalytic activity is required for the tumor response to RT (illustrated by green glare), it is largely dispensable for immune IRAK1 signaling. **(C)** “One-two punch” scenario, as afforded by a highly specific IRAK1 inhibitor with no activity against IRAK4. Such drugs would be expected to both blunt intrinsic tumor radioresistance (which depends on IRAK1 kinase activity) and spare IRAK1 mediated-antitumor immunity (which is less reliant on IRAK1 catalytic activity), resulting in a “one-two punch” on the tumor. The double-punch is illustrated by two red dart target symbols on the tumor. **(D)** “Double-edged sword” scenario, as afforded by a less specific IRAK1i with similar activity against IRAK4. Such IRAK1/4i would be expected to block both the tumor and immune responses to RT (each of which depends on IRAK4 catalytic activity; see text). Thus, in this scenario, intrinsic tumor radiosensitization activity would be retained but at the expense of blunting the immune component. A small, residual “punch” from the immune system on the tumor is indicated to further emphasize the detrimental effects of IRAK1/4i relative to the “one-two punch” effects of specific IRAK1i [compare with **(C)**]. Figure design by Ni-Ka Ford, printed with permission from with permission from ^©^Mount Sinai Health System.

While predominantly activated by microbes, IL-1R/TLR signaling is also notably engaged by stromal dendritic cells (DCs) and macrophages located in the vicinity of irradiated tumors (Figures 2A,B). Indeed, many of the molecules released by irradiated cancer cells (i.e., damaged/apoptotic/necrotic cancer cells) are bona fide ligands for IL-1R/TLR, including IL-1β itself and a number of DAMPs such as heat shock proteins, high mobility group protein 1 (HMGB1) and tumor DNA/RNA fragments (25–34). In response to IL-1R/TLR ligation, DCs engage in increased proliferation, maturation, and antigen presentation activity, ultimately triggering T-cell-mediated attacks of tumors located within and outside the radiation field (immune attacks of distant tumors are responsible for the “abscopal” effect of RT long observed in a small subset of patients). The molecules, immune cell types and mechanisms believed to underlie RT-induced, IL-1R/TLR-mediated antitumor immunity are briefly summarized in Figures 2A,B but have been extensively investigated and reviewed by expert colleagues (27–29, 31, 34–41).

The notion that RT acts as a trigger for IL-1R/TLR signaling is at the root of emerging RT+IT combination strategies making use of TLR agonists (TLRa) as adjuvant or neoadjuvant therapies (Figure 2A). TLRa such as CpG oligodeoxynucleotides (CpG-ODN, TLR9a) and various imidazoquinolines and nucleoside analogs (TLR7a; e.g., imiquimod/Aldara/R-837, resiquimod/R-848, DSR-6434, DSR-29133, 3M-011/854A) have demonstrated substantial efficacy, if not outright synergy, when combined with RT in mouse spontaneous or xenograft models of fibrosarcoma (38, 39), lymphoma (37), colorectal cancer (35, 36, 40), sarcoma (35), breast cancer (42), renal cell carcinoma (36), lung adenocarcinoma (43), pancreatic cancer (40), and metastatic osteosarcoma (36). Success with these preclinical studies has spurred a number of clinical trials of CpG-ODNs in combination with diverse chemo-RT treatment regimens (34, 44–46). Such trials initiated between 2015 and 2018 include NCT03410901, NCT01745354, NCT02254772, and NCT02266147 for the treatment lymphoma; NCT02927964 for the treatment of follicular lymphoma; NCT03322384 for the treatment of advanced solid tumors and lymphoma; and NCT03007732 for the treatment of prostate carcinoma [reviewed in (44)]. Despite mixed results so far, favorable clinical responses observed in patient subsets warrant further testing (34, 44–46).

## IRAK1 also Anchors an antiapoptotic Response to RT Distinct from IRAK1 Immune Signaling

As discussed in Introduction, while mammalian IRAK1 is a genuine protein kinase and is a central transducer in IL-1R/TLR signaling, its catalytic activity appears largely dispensable for innate immunity. TLR/IL-1R-independent roles for IRAK1 might explain this paradox, yet until recently no such non-immune IRAK1 function had been reported in vertebrates. In a screen for small molecules that restore RT-induced cell death in otherwise radioresistant *p53* mutant zebrafish (47, 48), we identified the microtubule inhibitor, oxfendazole (47). Surprisingly, target discovery identified IRAK1, and not tubulin, as the key target whose inhibition by oxfendazole was responsible for cell death recovery in irradiated fish (47). The requirement of IRAK1 for cell survival after RT was conserved in multiple human cancer cell lines tested in *vitro* or as tumor xenografts *in vivo*, regardless of *p53* genotype. Overexpression of IRAK1 was sufficient to force cell survival after RT in otherwise radiosensitive cells, in a manner that completely relied on its catalytic activity. Likewise, kinase-dead IRAK1 failed to complement *IRAK1* deficiency in both human and fish models (47). Rather than promoting survival through NF-κB and other attendant pathways, we found that IRAK1 acts to deny RT-induced apoptosis mediated by the PIDDosome complex (PIDD-RAIDD-caspase-2) (47, 49, 50). These observations identified an essential role for *IRAK1* outside of innate immunity as a gene required for the survival of irradiated vertebrate cells. IRAK1's reliance on its catalytic activity and engagement of a distinct antiapoptotic cascade were first clues that it might function in a pathway distinct from the canonical IL-1R/TLR axis (Figure 1B) (47).

Further evidence for IRAK1 functioning in a novel pathway came when we asked whether its known upstream proximal regulators, MyD88, IRAK4, and PIN1, were also required for the survival of irradiated cells. While IRAK4 and PIN1 clearly were, MyD88 clearly *was not*, whether in human cells or zebrafish embryos (47). Likewise, while IRAK4 and PIN1 were required for IRAK1 activation after RT, as assessed by T209 phosphorylation, MyD88 was not (47). In summary, RT-induced IRAK1 signaling differs from its canonical counterpart in three fundamental ways: (1) It fully relies on its kinase activity; (2) it acts through distinct downstream antiapoptotic mechanisms; and (3) it does not require MyD88 for activation by IRAK4 and PIN1 (Figure 1B vs. Figure 1A).

## IR-induced IRAK1 Signaling as a Driver of Intrinsic Tumor R-RT

Thus far, the case for IR-induced IRAK1 signaling acting as a driver of intrinsic tumor R-RT is four-fold. (i) IRAK1 and PIN1 are both sufficient to force R-RT in otherwise radiosensitive tumor cells (47). (ii) IRAK1 and PIN1 enzymatic activities are required for R-RT in cancer cell lines derived from multiple tumor types including HNSCC, breast cancer, colorectal cancer, and glioblastoma. These requirements for R-RT were verified *in vivo* in a mouse xenograft model of radioresistant HNSCC (47). (iii) IR-induced activation of IRAK1, as assessed by T209 phosphorylation, systematically correlated with tumor cell line sensitivity to RT+IRAK1i (47). (iv) Patients with high-risk HNSCC (HPV^neg^, mutant *TP53*) whose tumors resisted post-operative RT (51) show evidence of pathway activation, whereby elevated *PIN1* expression levels strongly associate with locoregional recurrence (LRR; *P* = 0.006) and reduced overall survival (OS; *P* = 0.007) (47). Notably, *PIN1* overexpression did not otherwise correlate with metastatic potential, arguing against the notion that PIN1 levels merely reflected an aggressive tumor subtype. While upregulation of *IRAK1* itself failed to correlate with R-RT in this cohort, this is not unexpected given the upstream role played by PIN1 in the pathway (see above; Figure 1B). Upregulation of PIN1 would in fact be expected to alleviate selective pressure to overexpress *IRAK1* in this context. Deregulation/amplification at the *IRAK1* locus might also not be a mechanism of choice via which tumors upregulate IRAK1 activity, though *IRAK1* overexpression has been detected in several tumor types (4, 19), with particularly convincing evidence for causality in triple-negative breast cancer (52). Alternative routes to IRAK1 activation include upregulation of upstream positive regulators, such as seen with PIN1 (see above) as well as S100A-7/9 proteins in breast cancers with 1q21.3 amplification (53); mutational inactivation or downregulation of negative regulators such as miR-146a, as seen in del(5q) acute myeloid leukemia (54); and likely additional mechanisms [reviewed in (4, 19)]. Complementing our microarray analyses with that of exome sequence datasets from radioresistant tumors across tumor spectra will further clarify the extent to which IR-induced IRAK1 signaling drives R-RT in human cancer.

## Targeting IRAK1 in Radioresistant Cancer

As discussed earlier, IRAK1 inhibitors (IRAK1i) were highly effective at suppressing R-RT in live *p53* mutant zebrafish and human cancer cell lines assayed *in vitro* or as mouse xenografts *in vivo* (47). Remarkably, effective doses of IRAK1i in these models caused little to no cell death in non-irradiated controls. This was in stark contrast with the traditional radiosensitizer cisplatin, which failed to overcome R-RT at maximal tolerable doses (47). This data, combined with the previously established viability of *Irak1*^−/−^ mice (55), suggests that systemic IRAK1i could restore RT sensitivity in patients without affecting healthy tissues outside of the radiation field.

While our work thus outlines a strong rationale for targeting IRAK1 in radioresistant tumors, as based on the projected efficacy and safety of such treatments, the strategy poses an immediate conundrum. Wouldn't systemic inhibition of the kinase simultaneously thwart the patient's immune attack on the irradiated tumor or the enhancement thereof by means of TLRa-based IT? Our tumor xenograft experiments, which were performed in immunodeficient mice, left this key question unanswered. Neoadjuvant administration of the TLRa (i.e., prior to RT+IRAK1i) or post-treatment delivery thereof might help circumvent the issue. However, our studies indicate that the window for IRAK1i radiosensitizing efficacy is limited to within a few hours of RT (47), and such treatments would thus be expected to come at the cost of blunting any acute immune contribution to the overall tumor response to RT.

However, such a “double-edged sword”-like tradeoff in efficacy is likely to be avoided by virtue of a critical, differential reliance of IRAK1 on catalytic activity when operating in response to IL-1R/TLR vs. when operating in response to RT (Figures 1A,B). As outlined earlier, kinase activity is essential for IRAK1 signaling in response to RT in all settings tested, both in zebrafish embryos and human cancer cells (47). In contrast, similar experiments making use of kinase dead IRAK1 variants in human cells (D340N, K239A) or knock-in mice (D359A) have indicated that catalytic activity is largely dispensable for IRAK1 function in IL-1R/TLR signaling (21–23, 56). Specifically, kinase dead IRAK1 retained full NF-κB inducing activity in all tested settings, presumably reflecting the protein's strict structural role when engaging TRAF6 (4, 24, 57). IL-1R/TLR-induced secretion of IL-6, TNFα, and IL-10 were likewise unaffected in bone marrow-derived macrophages from *Irak1*^*D*359*A*^ knockin mice (22). Thus, RT+IRAK1i-based radiosensitization strategies, whether alone or in combination with TLRa-based IT, would be expected to largely spare IL-1R/TLR-initiated immune attacks on the tumor, leading to an effective “one-two punch” both from within and outside the irradiated tumor (Figure 2C). It should be noted, however, that IRAK1 catalytic activity might not be entirely dispensable for all forms of IL-1R/TLR signaling. In the TLR7/9-IRF7 signaling branch, for instance, an intact IRAK1 kinase domain appears required for the transcriptional activation of IRF7 as well as for the timely induction of interferons by TLR7/9 (56), as further evidenced by a significant delay in IFN-β production by plasmacytoid DCs derived from *Irak1*^*D*359*A*^ mice (22). The relative contributions of the IL-1R/TLR-NF-κB (kinase-independent branch) vs. IL-1R/TLR-NF-α/β (partially kinase-dependent branch) to RT-induced antitumor immunity have not been rigorously explored to date and is an important topic for future studies.

The “one-two punch” hypothesis that IRAK1i will both intrinsically sensitize tumor cells to RT while also allowing for RT-induced antitumor immunity to proceed (Figure 2C) is further contingent on the use of IRAK1i that are highly specific to IRAK1. Indeed, unlike IRAK1, the catalytic activity of the sister kinase IRAK4 is essential for IRAK1 signaling in *both* the RT and IL-1R/TLR response pathways, in which IRAK4 acts to activate IRAK1 via direct phosphorylation on T209 (4, 13, 47). Thus, any IRAK1i with significant off-target activity against IRAK4 would be expected to radiosensitize the tumor proper but at the expense of affecting its immunogenic attack (Figure 2D). We recently confirmed the essential role of IRAK4 in RT-induced IRAK1 signaling *in vivo*, whereby (i) *irak4*-depleted *p53*^*MK*/*MK*^ zebrafish embryos recover RT-induced cell death as efficiently as *irak1*-depleted embryos (Liu and Sidi, unpublished observations); and (ii) *irak1*-depleted embryos reconstituted with T209A human *IRAK1* mRNA fail to resist RT-induced cell death, as opposed to embryos complemented with WT *IRAK1* mRNA (Li and Sidi, unpublished observations). Thus, IRAK1i used for radiosentization purposes should, at the very least, demonstrate marked selectivity for IRAK1 over IRAK4 (Figures 2C,D).

Of the many IRAK1i developed so far [reviewed in (19)], only one, pacritinib (58), combines clinical efficacy, acceptable safety, and selectivity for IRAK1 over IRAK4. This selectivity is only moderate, however, with IC50s of 6 and 177 nM vs. IRAK1 and IRAK4, respectively (19). In spite of IRAK1 and IRAK4 kinase domains sharing >90% amino-acid sequence identity within the ATP binding pocket as well as identical gatekeeper tyrosine residues, the selectivity—albeit moderate—of pacritinib for IRAK1 indicates that developing a highly specific IRAK1i is feasible in principle. The crystal structure of the human IRAK1 kinase domain bound to a small molecule was recently reported (10), which together with the known structure of the IRAK4 kinase domain (15) should help develop such selective IRAK1i. A very first example of such a compound was recently reported by Buhrlage, Treon, Gray and colleagues (59). The drug, Jh-X-119-01, labels IRAK1 at C302 and shows irreversible inhibition with an IC50 of 9.3 nM against IRAK1 vs. >10 μM vs. IRAK4. Disclosure of the structure of Jh-X-199-01 should spur future efforts to develop IRAK1i suited for use as radiosensitizers.

## Author Contributions

SS conceived the review and figures. SS and PL wrote the paper.

### Conflict of Interest

The authors declare that the research was conducted in the absence of any commercial or financial relationships that could be construed as a potential conflict of interest.
